# Risk of Developing Post-traumatic Stress Disorder in Severe COVID-19 Survivors, Their Families and Frontline Healthcare Workers: What Should Mental Health Specialists Prepare For?

**DOI:** 10.3389/fpsyt.2021.562899

**Published:** 2021-06-07

**Authors:** Marcin Sekowski, Małgorzata Gambin, Karolina Hansen, Paweł Holas, Sylwia Hyniewska, Julia Wyszomirska, Agnieszka Pluta, Marta Sobańska, Emilia Łojek

**Affiliations:** ^1^Department of Psychology, The Maria Grzegorzewska University, Warsaw, Poland; ^2^Faculty of Psychology, University of Warsaw, Warsaw, Poland; ^3^Department of Experimental Psychology, University College London, London, United Kingdom; ^4^Department of Psychology, Medical University of Silesia, Katowice, Poland

**Keywords:** coronavirus disease, post-traumatic stress disorder, severe acute respiratory syndrome, prolonged grief disorder, family, healthcare worker

## Abstract

Given the high mortality of the coronavirus disease 2019 (COVID-19), having severe COVID-19 may be a life-threatening event, especially for individuals at high risk of complications. Therefore, in the article we try to answer two questions that are relevant to public mental health: Can we define groups who are at higher risk of developing pandemic-related PTSD? How can health specialists prepare for it? Given the results of previous research on PTSD in epidemic (e.g., SARS) survivors, we suggest that mental health professionals in countries touched by the pandemic should prepare for an increase in the PTSD prevalence, specifically in: individuals who have had severe COVID-19; family members of these patients and of patients who have died; and frontline healthcare workers witnessing COVID-19 patients' sudden deaths, or numerous life-threatening situations. We postulate that these groups at risk should be routinely screened for PTSD in primary medical and pediatric care. Mental health services should prepare for providing therapeutic interventions for individuals with PTSD in the vulnerable groups, and support to their families, especially children.

## Introduction

The COVID-19 pandemic covered 223 countries, 136,291,755 individuals with confirmed infection and 2,941,128 deaths as of April 13, 2021 (1). Globally the case mortality rate from COVID-19 is 10.4 deaths per 100,000 inhabitants but it varies widely by region and country, with as high as 84.9 in Belgium, 67.5 in Andorra, and 64.3 in the United Kingdom (2). Age is the main risk factor for complications and death as a result of COVID-19; the disease death rate varies from 0.2% for individuals aged 10–39 years to 21.9% in people over 80 years. Death rate also increases in patients with pre-existing comorbid conditions, such as history of metastatic solid tumor (57%), myocardial infarction (47%), cerebrovascular disease (39%), congestive heart failure (37%), hemiplegia (34%), malignant neoplasm (27%), diabetes (20%), dementia (20%), chronic pulmonary disease (16%), hyperlipidemia (11%), and hypertension [8.4%; (3–5)]. Given the high COVID-19 mortality, the severe disease may be life-threatening and as such may be considered a traumatic event, especially for individuals at high risk of complications and death (6–8).

People who are exposed to traumatic events can develop post-traumatic stress disorder (PTSD). PTSD is a serious mental disorder that can develop in persons exposed not only to actual or threatened death, who directly experienced the traumatic event(s), but also in those who witnessed such an event personally, learned that the accidental or violent event occurred to a close family member, or friend or experienced repeated or extreme exposure to aversive details of the traumatic event personally. Such an event is criterion A for PTSD in the Diagnostic and Statistical Manual of Mental Disorders, Fifth Edition [DSM-5; (9)]. PTSD causes severe distress or impairment in psychosocial functioning and is marked by four main types of symptoms that last for at least a month, being, respectively, the criteria B, C, D, and E for the disorder: (i) intrusions associated with the traumatic event, e.g., involuntary distressing event-related memories, dreams, and flashbacks, or physiological reactions to cues that resemble the traumatic event; (ii) avoidance of event-related stimuli, i.e., internal (memories, thoughts, and feelings) or external (people, places, situations) reminders; (iii) negative alterations in event-related cognitions, and/or mood, e.g., inability to remember an important aspect of the event, distorted cognitions about the event leading to blaming himself or herself or others and/or persistent negative emotional state and inability to experience positive emotions, diminished activities, and/or detachment from others; and (iv) event-related increased arousal and reactivity, e.g., difficulty sleeping and concentrating, reckless or self-destructive behavior, and being easily irritated and angered (9). PTSD individuals are 2–5 times more at-risk of suicidal ideation, suicide attempt, and deaths by suicide (10). Previous research indicates that about 80% of individuals with PTSD have at least one other comorbid disorder, in particular: depressive and anxiety disorders, as well as substance use disorder (11, 12).

Given the exceptional epidemic situation we are facing now, can we define groups at high risk of developing pandemic-related PTSD? What steps can mental health specialists take to prepare for it? In the following paper we will briefly address these issues.

## What Groups Have High Risk of Developing Pandemic-Related PTSD?

We believe that mental health professionals in pandemic countries should be prepared for an increase in the PTSD prevalence in three specific groups during COVID-19 pandemic and later: (i) individuals who have had severe COVID-19 and who feared imminent death from the disease; (ii) family members of these severely touched patients or patients who have died as a result of the disease; and (iii) frontline healthcare workers (HCWs) witnessing COVID-19 patients' sudden deaths, or life-threatening situations.

### Severe COVID-19 Survivors

Among COVID-19 individuals, those with a severe course of the disease are particularly at risk of developing PTSD (6–8). Patients who had to undergo medical interventions to maintain or restore vital functions experience particularly intense traumatic stress (13, 14). A very traumatically stressed subgroup may also include individuals with severe disease who were refused health care as a result of health service failure during a pandemic (15, 16). The meta-analysis of 35 studies involving 79,170 people with COVID-19 (17) showed that almost a quarter of them (23%) develop a severe form of the disease (35 studies, 79,170 patients) which requires close monitoring. The total percentage of patients admitted to the intensive care unit was 11.0% (39 studies, 80,487 patients), with no significant differences between various countries or regions (17). The life of many of these individuals is threatened and many of them may experience high levels of fear of imminent death, which creates a traumatic event.

The effects of the current as well as of past epidemics on the mental health of survivors can help predict the psychopathological consequences of a severe life-threatening viral disease. Two Italian studies on recovered severe COVID-19 survivors reported that the prevalence of PTSD is 10.4% (8) and 30.2% (7), respectively. Additionally, in survivors of the severe acute respiratory syndrome (SARS) during the epidemic that covered 29 countries in 2002/2003, infecting 8,096 individuals, of whom 774 died PTSD was present in 39% of patients 10 months after discharge (18), and over time the frequency of PTSD even increased, ranging from 42 to 54.5% after 31–51 months from discharge (18–20). Given that an estimated 3.6% of U.S. adults had PTSD in the former year, and the lifetime prevalence of this syndrome being 6.8% (21), the cited studies indicate that PTSD is a common chronic mental health problem following a virus disease with high mortality (22).

It should be added that COVID-19 infected persons most often develop less severe symptoms such as fever (78.8%), loss of smell and/or taste (from 19.0 to 73.6%), cough (53.9%) and malaise (37.9%), and sometimes diarrhea (9.5%), rhinitis (7.5%), and abdominal pain and vomiting (4.5%) (17, 23, 24) which are usually not life-threatening and are treated at home with over-the-counter medications. Infection may also be asymptomatic (25–27). Such cases are not likely to develop PTSD as COVID-19 infection in most of the cases does not lead to symptoms severe enough to be life threatening and these individuals do not meet the A criterion for PTSD (9). However, some persons, although not hospitalized, may experience severe symptoms of COVID-19 and fear of imminent death, thereby experiencing a traumatic event, and can develop PTSD; high levels of fear of death during the event, not the type of medical treatment, are the hallmark of a traumatic stressor.

### Families of Severe COVID-19 Survivors and of Persons Who Died of the Disease

The second group at risk of developing pandemic-related PTSD are the families of people whose lives were at risk due to severe COVID-19 as well as the families of those who died of the disease under emergency circumstances. Being a witness to the threat to the life of another person, especially a loved one in the course of COVID-19, can be a traumatic event (9). The family's situation becomes even more complicated when a loved one has died in the course of the disease. Bereaved persons can then be obliged not only to deal with the traumatic events of life-threatening viral disease and the death of a loved one, but also with intense grief symptoms. The sudden death of a loved one and the inability to prepare for the death—which often occurs during a pandemic—is a risk factor for prolonged grief disorder (28). Grief of family members may be additionally difficult and complicated due to the circumstances surrounding death and mourning during a pandemic, e.g., quarantine of the dying persons, and delayed and very modest funerals. Grief and mourning in this time, due to accumulation of death and difficulties with recognizing individual bereavement and pain during pandemic, especially after loss of an elderly with chronic diseases, can be disenfranchising and therefore additionally complicated (29).

### Frontline Healthcare Workers

The third group at risk of developing PTSD during a pandemic are healthcare professionals who experience traumatic stress in the context of witnessing patients' dying and their lives being threatened, especially in cases of insufficient or inadequate intensive medical equipment. Furthermore, shortages of protective or medical equipment (30, 31) can be potentially traumatic if they lead the HCWs to fear of contamination and own imminent death. Own severe course of the disease and being a witness to the threat to life or death of other people may lead to the accumulation of traumatic stress in HCWs. Studies showed that after the SARS epidemic in 2002/2003, PTSD developed more often in infected HCWs than in non-HCWs (20, 32). We may predict that other professional groups such as employees of nursing homes and of other long-term care facilities also may experience some of these above mentioned traumatic stress factors (33).

## What Steps Can Be Taken to Prepare for Pandemic-Related PTSD?

A systematic approach to services to prevent, diagnose, and treat PTSD in risk groups is needed (34). First of all, mental health experts should be aware of the risk of developing PTSD in severe COVID-19 survivors who feared imminent death from the disease, their family members and family members of patients who have died as a result of the pandemic, as well as frontline HCWs witnessing patients' sudden deaths, or life-threatening situations. Information about PTSD and its symptoms should be made available to all HCWs and to mental health patients and their families during and in the months following the pandemic.

Second, we postulate that the three groups at risk described above should be routinely screened for PTSD in the months following the onset of the disease as the usual window of PTSD onset lies between 1 and 6 months after a traumatic event. Screening should be done in primary medical and pediatric care if resources are immediately available for timely follow-up by mental health professionals with specific expertise in PTSD assessment, and—in optimal circumstances—treatment of this disorder. Screening should distinguish between other mental health problems vs. PTSD. Measurement should begin with screening first for traumatic events, i.e., directly experiencing or witnessing death or imminent threat of death due to the pandemic (or other concurrent or previous incidents) being criterion A of the disorder according to DSM-5. PTSD symptoms should be screened only if potentially traumatic events are identified. For example, non-symptomatic individuals who test positive for COVID-19 are not at risk and should not be screened for PTSD, although they may develop other mental disorders such as depressive or anxiety disorder (35). Co-occurrence of PTSD and other disorders [see (7, 8)] is associated with high levels of distress, burden and reduced quality of life (11, 12), thus special clinical attention should be paid to individuals with PTSD experiencing additional mental health problems.

Bereaved surviving close ones who died unexpectedly as a result of the pandemic should be screened not only for PTSD but also for prolonged grief disorder, for example with the Prolonged Grief Disorder-13-Revised scale (36). PGD measurement should be carried out 12 months after the loss, as—according to DSM-5-TR—this is the time after which the persistence of severe grief symptoms, if they interfere with the psychosocial functioning, indicates the presence of this disorder (37).

Third, mental health services should prepare to provide evidence-based therapeutic interventions for individuals with PTSD such as prolonged exposure therapy, and cognitive processing therapy (38). It would be worth training clinicians in various countries in delivering these evidence-based interventions online, as well as to test their effectiveness in individuals with pandemic-related PTSD. Moreover, mental health professionals should be encouraged to: (i) share their experiences on the adjustments to be made in order to tailor these interventions to the current situation, e.g., necessity to provide interventions online and to acknowledge the specificity of the clinical picture of symptoms in severe COVID-19 survivors and their families (including the bereaved), and HCWs; (ii) define frameworks to effectively implement evidence-based interventions in mental health services in various countries (in particular in low income countries); and (iii) prepare accessible and easy-to-follow online trainings for professionals from various countries on evidence-based therapeutic interventions for PTSD.

Finally, studies indicate that the severity of PTSD symptoms in parents is associated with an increase in behavioral and emotional problems in their children (39, 40). Therefore, one should be prepared to prevent such cascading effects of the pandemic by considering psychological support for children of parents who have developed PTSD due to the pandemic. Again, one of the challenges would be providing support for children and their families through online services.

The issue of the full prevalence of pandemic-related PTSD remains a matter of future research as symptoms may develop up to 6 months after the traumatic event. Research showing the full scope of pandemic-related PTSD prevalence will be possible only a few months after the end of the outbreak. Former studies indicated a large prevalence of PTSD in survivors of the earlier coronavirus epidemic (SARS), especially in HCWs, therefore we suggest that mental health professionals should prepare for the frequent occurrence of PTSD in this high risk groups. Families of survivors of severe diseases with high mortality and those who died from the pandemic, as well as frontline HCWs who were not infected but have witnessed numerous deaths and life-threatening situations—although not included in such systematic studies as survivors—may also experience traumatic stress during the outbreak and develop PTSD. Notwithstanding, we suggest that health care policies should consider routine screening for the presence of PTSD symptoms in the three groups at risk described in this paper, together with preventive and treatment strategies of PTSD, and related risks such as suicide.

## Author Contributions

MS wrote the first draft of the manuscript. All authors edited it, wrote parts of the reviewed manuscript, and accepted its final form.

## Conflict of Interest

The authors declare that the research was conducted in the absence of any commercial or financial relationships that could be construed as a potential conflict of interest.
